# Enhanced Mechanical and Thermal Properties of Epoxy Resins Through Hard–Soft Biphasic Synergistic Toughening with Modified POSS/Polysulfide Rubber

**DOI:** 10.3390/polym18020184

**Published:** 2026-01-09

**Authors:** Xi Yuan, Zhineng Tan, Shengwen Liu, Hang Luo, Zhuo Chen, Dou Zhang

**Affiliations:** 1 College of Chemistry and Chemical Engineering, Central South University, Changsha 410083, China; xiyuan@csu.edu.cn (X.Y.); 232311082@csu.edu.cn (Z.T.); 2 State Key Laboratory of Powder Metallurgy, Central South University, Changsha 410083, China; shengwenliu@csu.edu.cn (S.L.); hangluo@csu.edu.cn (H.L.)

**Keywords:** mechanical performance, epoxy/POSS hybrid materials, amine-epoxy click reaction, curing kinetics, synergistic toughening

## Abstract

Toughening modification of epoxy resin (EP) matrices is important for advancing high-performance fiber-reinforced composites. A promising strategy involves the use of multi-component additive systems. However, synergistic effects in such additive systems are difficult to achieve for multidimensional performance optimization due to insufficient interfacial interactions and competing toughening mechanisms. Herein, a “hard–soft” biphasic synergistic toughening system was engineered for epoxy resin, composed of furan-ring-grafted polyhedral oligomeric silsesquioxane (FPOSS) and liquid polysulfide rubber. The hybrid toughening agent significantly enhanced the integrated performance of the epoxy system: Young’s modulus, tensile strength, and elongation at break increased by 13%, 56%, and 101%, respectively. These improvements are attributed to the formation of enriched molecular chain entanglement sites and optimized dispersion, facilitated by nucleophilic addition reactions between flexible rubber segments and rigid FPOSS units with the epoxy matrix. The marked enhancement in toughness primarily stems from the synergistic toughening mechanism involving “crazing pinning” and “crazing-shear band”. Concurrently, FPOSS incorporation effectively modulated the curing reaction kinetics, rendering the process more gradual while substantially elevating the glass transition temperature (T_g_) of the cured system by 16.82 °C and endowing it with superior thermal degradation stability. This work provides a simple and unique strategy to leverage multi-scale mechanisms for the construction of epoxy-based composites with good toughness and strength, and enhanced heat resistance.

## 1. Introduction

Epoxy resin (EP) has been widely used as the matrix of high-performance fiber reinforced composites due to its excellent mechanical properties, superior fiber adhesion, dimensional stability, outstanding insulation properties, and chemical resistance [1,2,3]. However, similar to most thermosetting materials, the highly crosslinked network structure formed upon curing imparts inherent brittleness and poor impact resistance to epoxy resins [4]. To overcome this limitation, various toughening agents, including nanoparticles [5,6,7,8], rubber elastomers [9], core–shell particles [10,11], and thermoplastic polymers [12], are frequently incorporated as dispersed phases or comonomers to improve the toughness of epoxy resin [13]. Unfortunately, the single toughening modification also leads to detrimental compromises in other critical performance aspects [13,14]. For example, the addition of rubber elastomer usually decreases the thermal stability and/or strength of the modified EP [15,16]. Similarly, inorganic nanoparticles tend to agglomerate and undergo macrophase separation in EP matrices due to their high surface energy and significant incompatibility [17], which results in unfavorable stress concentration, thereby decreasing mechanical performance and processability of the cured EP [18,19]. Therefore, achieving effective toughening of EP while preserving or even enhancing its thermal and mechanical properties remains a significant challenge.

In this case, many multi-component additive systems are explored to enhance the comprehensive performance of epoxy resin [20,21]. Thomann et al. added organophilic layered silicates and polyether liquid rubbers to modify the epoxy matrix [15]. The stearate compatibilizer was used as the second phase to achieve phase separation of the rubber, which improved toughness and only reduced stiffness by 10%. Tsang et al. incorporated both silica nanoparticles (SiO_2_) and core–shell rubber particles (CSR) into the epoxy resin and observed that the fracture behavior of the hybrid SiO_2_/CSR particle system was close to the single-particle systems, showing no significant synergistic enhancement [22]. This phenomenon was attributed to the substantial size disparity between CSR particle agglomerates and SiO_2_ nanoparticles, which led to insufficient interfacial interactions. The simplistic physical blending of toughening agents frequently fails to achieve the synergistic toughening effect, and even decreases the mechanical properties of the epoxy matrix [15,23]. This unsatisfactory performance of modified EP via multi-component additive method can be attributed to the intrinsic contradictions between different toughening mechanisms and absence of robust interface bonding between toughening components [22,24].

Toughening nanofillers featuring chemical bonding capability to the epoxy resin matrix were developed as an effective strategy to mitigate the limitations of inadequate interfacial interactions and competition of toughening mechanisms. The core structure of polyhedral oligomeric silsesquioxane (POSS) features an inorganic cage-like skeleton composed of Si-O-Si linkages [25]. Organic functional groups are attached at the silicon vertices, yielding three-dimensional cage molecules with nanoscale dimensions [26]. The diversity of its organic substituents enables high design flexibility, promoting excellent reactivity and compatibility with epoxy resin matrices. The POSS tends to form microphase-separated domains within the EP matrices due to its precise nanoscale architectures and unique organic–inorganic hybrid nature [27,28,29]. Furthermore, its highly ordered molecular structure provides exceptional strength and hardness, while the inorganic silica framework imparts outstanding thermal stability [30]. Therefore, these characteristics render POSS a highly promising material for reinforcing and toughening EP [31,32]. However, POSS tends to undergo agglomeration due to its high surface energy [33], severely restricting its toughening effect on epoxy resin matrices [34]. Consequently, extensive research focused on modifying POSS to mitigate agglomeration and enhance interfacial bonding, thereby improving dispersion and composite performance [34,35,36]. Therefore, modified POSS can enhance interfacial adhesion through chemical bonding with the epoxy network and serve as a nano-trigger site to facilitate more effective stress transfer, which makes it highly desirable in multi-component toughened modification of EP.

Based on the above considerations, this study constructs a “hard–soft” biphasic synergistic toughening system that enables effective multi-scale energy dissipation and simultaneously enhances both the strength and toughness of EP. Specifically, the hybrid toughening agent consists of modified polyhedral oligomeric silsesquioxane (FPOSS) and liquid polysulfide rubber (PSR). Liquid polysulfide rubber serves as the primary reactive toughening component, inducing phase separation to enhance the overall plastic deformation capability of epoxy. Simultaneously, furan-ring-modified octaepoxy POSS (FPOSS) was synthesized via a mild amine–epoxy click reaction. This FPOSS participated chemically in the epoxy resin curing reaction, ensuring excellent compatibility and reactivity within the Epoxy/PSR system. When employed as a composite toughening agent alongside PSR to modify the epoxy resin, FPOSS overcame the intrinsic contradiction in the toughening mechanism of simplistically physically blended multi-component toughening agents. This strategy facilitated more effective stress transfer from the matrix to the POSS nanoparticles. Furthermore, the chemical bonding between FPOSS and the epoxy network generates a robust interface reinforcement effect, allowing FPOSS to be firmly anchored within the cured structure. This design aimed to achieve simultaneous enhancements in both mechanical strength and toughness. The curing behavior and thermal properties of the epoxy resin system were systematically investigated. Results demonstrated that the modified POSS acted as an “anchor point” within the epoxy network, confining polymer chain movement and consequently increasing the glass transition temperature (T_g_). Additionally, its excellent char-forming ability significantly enhanced the thermal stability of the epoxy resin.

## 2. Materials and Methods

### 2.1. Raw Materials

N, N-Diglycidyl-4-glycidyloxyaniline (coded as AFG-90), a light yellow transparent viscous liquid with an epoxy equivalent weight = 90–105 g/equiv, and the curing agent (4,4′-Diaminodiphenyl sulfone, coded as CA-15) were purchased from Guangzhou Tai Ji New Materials Co., Ltd. (Guangzhou, China). Liquid Polysulfide Polymers (coded as PSR, LP-3), a light amber liquid with an average molecular weight of 1000), were obtained from Toray Fine Chemicals Co., Ltd. (Tokyo, Japan). 3-Glycidyloxypropyltrimethoxysilane (KH-560, purity > 97%) and furfurylamine (FA, purity > 99%) were purchased from Shanghai Aladdin Bio-Chem Technology Co., Ltd. (Shanghai, China). Hydrochloric acid (AR) and sodium sulfate (AR) were purchased from Sinopharm Chemical Reagent Co., Ltd. (Shanghai, China). Methyl alcohol (purity > 99.5%) and dichloromethane (purity > 99.5%) were purchased from Shanghai Titan Scientific Co., Ltd. (Shanghai, China). Unless otherwise mentioned, all reagents were used as received without further purification. The chemical structures of AFG-90, PSR, and KH-560 are shown in Figure S1.

### 2.2. Synthesis of Octaepoxysilsesquioxane (EP-POSS)

**Figure 1:**
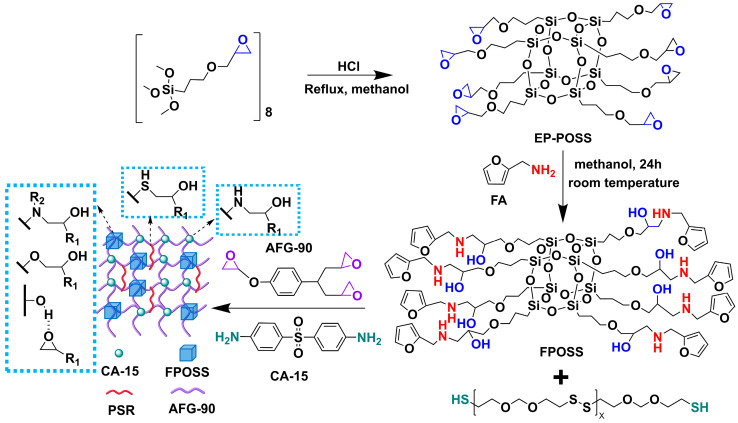
Synthesis process of epoxy/PSR/FPOSS nanocomposite.

EP-POSS (Octaepoxysilsesquioxane) was synthesized by a modified procedure according to previous literature [37]: 30 mL concentrated HCl of 37 wt.% and 300 mL methanol were added to a 500 mL flask equipped with a magnetic stirrer (Shanghai Titan Scientific Co., Ltd., Shanghai, China). 15 mL 3-Glycidyloxypropyltrimethoxysilane (KH-560) and 60 mL methanol were mixed in a constant pressure dropping funnel. The methanol solution of KH-560 was slowly added dropwise with stirring and completed within 1 h under nitrogen atmosphere. After the addition was complete, the mixture was refluxed at 90 °C for 24 h to ensure the termination of hydrosilylation reaction (Figure 1). The solvent was removed by rotary evaporation, and the crude product was washed with cold MeOH three times to remove the unreacted KH-560. Then, the resulting viscous paste was dissolved in dichloromethane and then washed three times with deionized water. The organic layer was then dried over anhydrous sodium sulfate. The residue was purified by column chromatography to give the compound EP-POSS as a colorless, transparent, viscous and flowable liquid in 80% yield.

### 2.3. Synthesis of FPOSS

A total of 1.2 g EP-POSS (0.9 mmol) was dissolved in 15 mL of methanol. Then, 0.7 g furfurylamine (FA, 7.2 mmol) was subsequently added dropwise at room temperature to the solution through a constant pressure dropping funnel and the reaction mixture was stirred at room temperature for 24 h (Figure 1). The product was filtered and washed repeatedly with methanol and deionized water. The combined filtrate was rotary-evaporated and then dried in a vacuum oven at 60 °C to afford a pale yellow powder, coded as FPOSS (1.71 g, 90% yield).

### 2.4. Preparation of PSR Toughened Epoxy Resin

Firstly, a certain amount of EP and different amounts of PSR (5/10/15/20/25/30 wt.% of the total weight of EP) were mixed thoroughly with a magnetic stirrer until a transparent and homogeneous mixture was obtained and the mixture pre-cured at 150 °C for 1 h. After the mixture cooled to room temperature, the curing agent, CA (epoxy group:amino-group = 1:1), was added and stirred thoroughly until uniform at 25 °C for 5 min to obtain a clear liquid. Then, the homogenized mixture was degassed under vacuum at 25 °C for 20 min and was quickly transferred to a silicone mold which had been preheated at 50 °C, followed by curing using the procedure of 90 °C/2 h + 110 °C/2 h + 160 °C/2 h. Finally, the mold was naturally cooled down to room temperature to obtain a cured resin with specific shapes suitable for mechanical tests, coded as E/P-x, where x = 5, 10, 15, 20, 25 and 30, respectively, representing the mass percentage of PSR. The detailed formulation is listed in Table S1. Similarly, pure EP was prepared and cured using the procedure of 90 °C/2 h + 110 °C/2 h + 140 °C/2 h to obtain the cured resin, coded as EP.

### 2.5. Preparation of POSS-Modified Epoxy Resin Nanocomposite

Preparation procedure similar to the E/P system: a certain amount of the EP/PSR hybrids (EP:PSR = 100:20) and different amounts of FPOSS (pre-dissolved in acetone) were mixed thoroughly under mechanical stirring and pre-cured at 60 °C for 1 h. After cooling to room temperature, the curing agent, CA (epoxy group:amino-group = 1:1), was added to the mixture. This mixture was then cured using the procedure of 80 °C/2 h + 120 °C/2 h + 150 °C/2 h to obtain the cured resin, coded as E/P/F-x, where x = 1, 3, 6, 10, respectively, representing the mass percentage of FPOSS. The compositions for the various resin systems are listed in Table S1. Similarly, EP and FPOSS (3 wt.% of the total weight of EP) were mixed thoroughly under mechanical stirring and using the procedure of 80 °C/2 h + 120 °C/2 h + 150 °C/2 h to obtain the cured resin, coded as E/F.

### 2.6. Characterization

^1^H NMR, ^13^C NMR and ^29^Si NMR spectra of the monomers were obtained using a Bruker Avance III 400 MHz spectrometer (Bruker Corporation, Karlsruhe, Germany) with CDCl_3_ or DMSO-d_6_ as the solvent and tetramethylsilane (TMS) as an internal reference. Fourier transform infrared (FTIR) spectra were recorded on a Nicolet iN10 spectrometer (Thermo Fisher Scientific, Waltham, MA, USA) over the wavenumber range of 400–4000 cm^−1^. The molecular weight of monomers was measured by high resolution mass spectrometry (HRMS) with a Bruker ESI-Q-TOF MS/MS Mass Spectrometry (Bruker Corporation, Karlsruhe, Germany). X-ray photoelectron spectroscopy (XPS) analysis was performed using a Thermo Scientific K-Alpha spectrometer (Thermo Fisher Scientific, Waltham, MA, USA). The rheological measurements of the sample were performed on an MCR 302e (Anton Paar, Graz, Austria) using a 25 mm diameter parallel plate with a gap size of 1 mm. A strain amplitude of 1% was applied. Isothermal time sweep tests were performed at 25 °C with a vibrational frequency of 10 rad/s. Particle size distribution was measured by Dynamic Light Scattering (DLS) with a Malvern Zetasizer Nano ZS90 (Malvern Instruments, Great Malvern, UK). E/P/F-3 hybrid was diluted with ethanol and dispersed ultrasonically for 5 min. Each sample was measured in triplicate. Differential scanning calorimetry (DSC) curves of the prepolymers were conducted on a TA Discovery DSC25 (TA Instruments, New Castle, DE, USA) under a nitrogen atmosphere at a heating rate of 5, 10, 15 or 20 °C min^−1^ in the temperature range between 30 and 200 °C. In all measurements, the glass transition temperature (T_g_) of the samples was determined from the second heating scan. Thermogravimetric analysis (TGA) was performed on a TA Discovery TGA 550 (TA Instruments, New Castle, DE, USA) under a nitrogen or air atmosphere with a flow rate of 20 mL min^−1^. The samples were heated over the range from 30 °C to 600 °C with a heating rate of 10 °C min^−1^.

Flexural properties of the resins were evaluated using a universal material test machine (Instron, 3369, Canton, OH, USA) at room temperature. The dimensions of each sample were (80 ± 2) mm × (10.0 ± 0.2) mm × (4.0 ± 0.2) mm, and the span length was 64 mm. The test speed was set to be 1 mm/min. The final flexural data were calculated as the average of five effective measurements. Tensile properties of each resin were obtained on a universal material test machine (Instron, 3369, Canton, OH, USA) at room temperature. The sample dimensions were (170 ± 2) mm × (20.0 ± 0.2) mm × (4.0 ± 0.2) mm. The test speed was set to be 1 mm/min. The final tensile data were calculated as the average of five effective measurements. The micromechanical properties of the resins were carried out on a nanoindenter instrument (UNHT^3^, CSM Products, Inc., Peseux, Switzerland). The loading and unloading rates were 0.05 s^−1^, and the maximum indentation force of 50 mN was kept for 30 s. At least 6 impressions were made on each sample with a distance of 100 μm between impressions to avoid interaction. Scanning electron microscopy (SEM, TESCAN, Brno, Czech Republic) experiments and energy dispersive spectroscopy (EDS) were used to observe the fracture surfaces and residual chars of the samples at an accelerating voltage of 15 kV.

## 3. Results and Discussion

### 3.1. Synthesis and Characterization of EP-POSS

**Figure 2:**
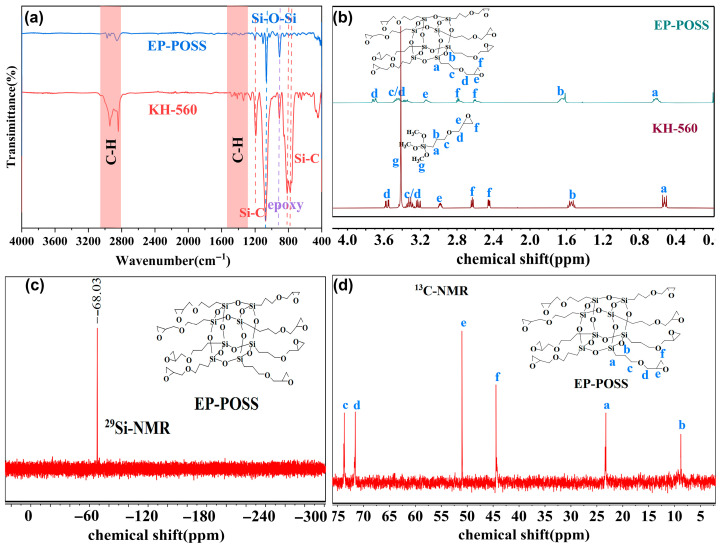
(**a**) FT-IR spectrum of KH-560 and EP-POSS; (**b**) ^1^H NMR spectra of KH-560 and synthesized EP-POSS; (**c**) ^29^Si NMR spectrum and (**d**) ^13^C NMR spectrum of EP-POSS.

The reaction pathway for synthesizing EP-POSS via hydrolysis and condensation of KH-560 is depicted in Figure 1. The structure of EP-POSS was characterized by FT-IR, NMR, HRMS, and XPS. Figure 2a presents the FT-IR spectra of KH-560 and EP-POSS. Characteristic absorption peaks at 1104 cm^−1^ and 1074 cm^−1^ are ascribed to the asymmetric stretching vibrations of Si-O-Si within the T_8_ cage structure [38]. The presence of epoxy groups is confirmed by the peak at 910 cm^−1^, while vibrations of Si-C bonds are evidenced by absorptions at 1250 cm^−1^, 813 cm^−1^, and 776 cm^−1^ [37]. Furthermore, the absence of broad absorption bands near 3500 cm^−1^ indicates that no detectable Si-OH groups are present, confirming the completion of the silane condensation reaction. The stacked ^1^H NMR spectra of EP-POSS and KH-560 are presented in Figure 2b. The spectrum exhibits characteristic signals corresponding to the γ-(2,3-epoxypropyl) propyl side chain, confirming the epoxy group remains intact without ring-opening. Critically, no characteristic peak attributable to the silanol group was observed at δ = 1.9 ppm, indicating completion of the condensation reaction. The methoxy proton peak (δ = 3.42 ppm) present in the monomer had disappeared in the product spectrum, confirming consumption of the methoxy group during the reaction and the absence of residual monomer in the purified product. Additionally, distinct shifts in the chemical shifts of protons adjacent to functional groups provide further evidence for the formation of a T_8_ cage-like structure and epoxy-functionalized POSS. The ^29^Si NMR spectrum (Figure 2c) exhibits a single resonance at δ = −68.5 ppm, signifying that all silicon atoms within the product possess an identical chemical environment. According to literature reports [39,40], this singular resonance is assigned to the characteristic peak of the T_8_ cage-like structure. Furthermore, the ^13^C NMR spectrum (Figure 2d) displays carbon signal peaks consistent with the carbon atoms of the organic substituents at the vertices of the EP-POSS monomer. Significantly, no methoxy carbon signals connected to silicon were detected, demonstrating complete hydrolysis and condensation of KH-560.

The HRMS spectrum of EP-POSS (Figure S2) shows the experimental [M+Na]^+^ value is 1359.3508, respectively, which closely matches the theoretical value of 1359.3508 (T_8_). To further characterize EP-POSS, XPS analysis was performed. Peak fitting of the C 1s signal (Figure S3a) revealed contributions from C-O (286.91 eV), C-C (284.80 eV), and C-O-C (288.26 eV) bonds [41]. Similarly, the O 1s signal was deconvolved into peaks attributable to C-O-C (532.71 eV) and Si-O-Si (531.82 eV) bonds [42]. Notably, no characteristic peak attributable to hydroxyl groups (C-OH, typically expected around 532.00 eV) was observed in the spectrum (Figure S3b), suggesting that the epoxy group remained intact without a ring-opening reaction. Taken together, these characterization results are consistent with the expected chemical structure of EP-POSS, confirming its successful synthesis.

### 3.2. Characterization of FPOSS

**Figure 3:**
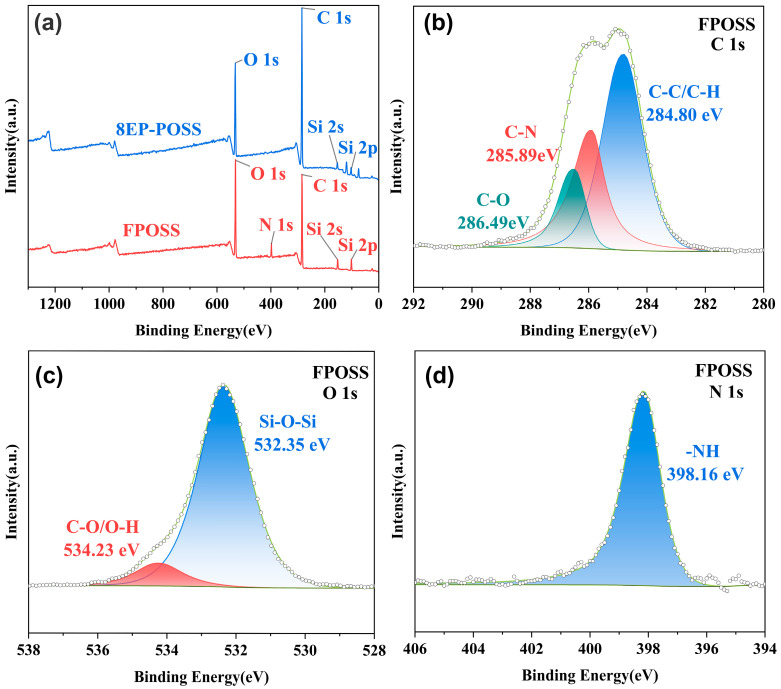
(**a**) XPS spectra of EP-POSS and FPOSS; High-resolution XPS spectra of C1s (**b**), O1s (**c**) and N1s (**d**).

To enhance reaction efficiency, EP-POSS was modified via an amine–epoxy click reaction at room temperature. The formation of the reaction product, FPOSS, was confirmed through various characterization techniques. Figure 3a presents the XPS spectra of EP-POSS and FPOSS, with the corresponding surface element molar fractions summarized in Table 1. Compared to EP-POSS, the spectrum of FPOSS exhibits a new characteristic peak at 398.16 eV assigned to N1s signal. Furthermore, the data in Table 1 reveal a significant increase in carbon content and a substantial decrease in oxygen content for FPOSS. These results confirm the successful linkage between EP-POSS and furfuryl amine via the amine–epoxy click reaction. Figure 3b displays the C1s fine spectrum of FPOSS. Notably, the characteristic peak attributed to the epoxy group’s C-O-C bond (288.26 eV) present in EP-POSS, is absent in FPOSS. The O1s fine spectrum (Figure 3c) is fitted into peaks corresponding to C-O/C-OH (534.23 eV) and Si-O-Si (532.35 eV). The N1s fine spectrum (Figure 3d) shows only a single absorption peak at 398.16 eV, assigned to secondary amine. The appearance of the hydroxyl characteristic peak, coupled with the disappearance of the primary amine characteristic peak, confirms the occurrence of the epoxy ring-opening reaction and its completeness. In addition, FTIR and NMR spectra of FPOSS confirmed that the successful grafting of FA onto the EP-POSS molecular structure to form the target ring-opened adduct, with no residual free primary amino or epoxy groups detected (see S3 Section of the Supporting Information). Through a pre-curing process, flexible rubber segments and low-content FPOSS were grafted onto the main chain of EP. FT-IR analysis of samples E/P-25 and E/P/F-3 (Figure S4c) revealed a significant increase in the intensity of the O-H characteristic peak at 3490 cm^−1^ upon incorporation of polysulfide rubber (PSR) and FPOSS. This is attributed to reactions between the thiol groups (-SH) in PSR, the secondary amino groups (-NH-), and hydroxyl groups (-OH) in FPOSS with the epoxy groups.

Thermogravimetric analysis (TGA) results (Figure S5) indicate that FPOSS exhibits an initial decomposition temperature (T_5%_) of approximately 304 °C and a maximum weight loss rate temperature (T_max_) of approximately 358 °C. The char yield at 600 °C is approximately 38.41%, demonstrating that it possesses excellent thermal stability and superior carbonization capability. It is well established that uniform dispersion of rigid fillers within the resin matrix is critical for achieving high fracture toughness [43,44]. The dispersion state of FPOSS in the epoxy system was further evaluated by particle size distribution analysis (Figure S6). The distribution exhibited a sharp peak centered at approximately 1.896 nm, accompanied by a narrow size range and low polydispersity. This indicates that FPOSS can be uniformly distributed in the resin pre-polymer, thus avoiding macroscopic phase separation caused by initial agglomeration. Such molecular-scale dispersion at the initial stage serves as a crucial prerequisite for achieving homogeneous nanocomposite structures and lays a fundamental foundation for maintaining molecular-level dispersion in the cured resin. This behavior is mainly attributed to the covalent bonding between FPOSS and the epoxy matrix, along with their intrinsic compatibility.

### 3.3. Curing Behaviors and Mechanism

**Figure 4:**
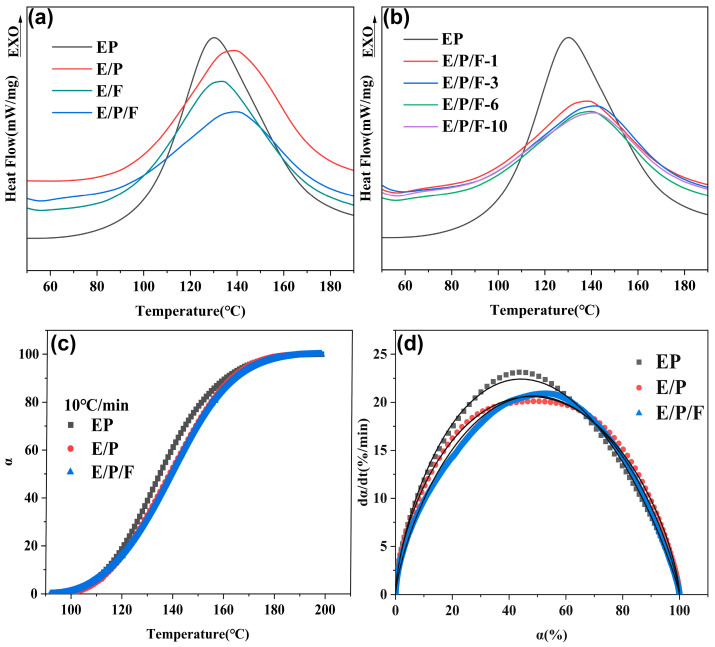
(**a**) DSC thermograms of epoxy system with different toughening agents at heating rate of 10 °C/min; (**b**) DSC thermograms of pure EP and E/P/F-x mixtures at heating rate of 10 °C/min; (**c**) The relationship between curing degree α and temperature at heating rate of 10 °C/min; (**d**) Evolution of the curing rate dα/dt with α. The solid line is the result of the fit of Equation (1).

The curing behavior of the EP, E/P, E/F, and E/P/F systems was investigated using non-isothermal DSC to examine the differences and determine the optimal curing process. As shown in Figure 4a,b, the DSC curves of the different epoxy curing systems all exhibited a single exothermic peak, corresponding to the ring-opening polymerization between the epoxy and amine groups. The single peak shape indicated that the cured system is miscible and homogeneous, with no macroscopic phase separation. As the FPOSS content increased, the peak value of the curing exothermic peak decreased, the peak broadened, and its sharpness diminished (Figure 4b). This indicates that FPOSS effectively moderates the reaction kinetics by reducing the overall heat release and promoting a more gradual curing process. At a constant heating rate of 10 °C/min, the curing degree (α) of all systems as a function of temperature (T) displayed a characteristic S-shaped curve (Figure 4c) [45]. Figure 4d depicts the curing rate (dα/dt) plotted against α for the EP, E/P, and E/P/F systems. All three exhibited comparable profiles, where dα/dt initially increases before decreasing. The maximum curing rates occurred at α = 0.44 (EP), α = 0.49 (E/P), and α = 0.52 (E/P/F), respectively. Notably, the E/P/F system attained its maximum curing rate at a higher conversion and exhibited a lower peak dα/dt value compared to the EP system. This indicated a more moderate reaction process for epoxides within the E/P/F system [46], and this relatively gradual evolution of reaction rate contributed to enhanced uniformity throughout the cured matrix.

Kamal and Sourour proposed a self-catalytic reaction model to describe the curing kinetics of epoxy resins, expressed as [47]:

(1) $$\frac{d_{\alpha }}{d_{t}}=\left(k_{1}+k_{2}a^{m}\right){\left(1-\alpha \right)}^{n}$$

where *m* and *n* are independent reaction orders, α represents the degree of curing, and the fitting parameters *k*_1_ and *k*_2_ denote the curing rate constants for the non-catalytic and self-catalytic reactions, respectively. As shown in Figure 4d, the experimental data exhibited excellent agreement with the Kamal–Sourour model (R^2^ > 0.99). Kinetic parameters (*k*_1_, *k*_2_, *m*, *n*) obtained via nonlinear least-squares fitting using MATLAB (R2024a) are summarized in Table 2. The *k*_2_ values significantly exceeded *k*_1_ across all systems, confirming that the self-catalytic reaction proceeds substantially faster than the non-catalytic reaction. Compared with the pure EP system, both *k*_1_ and *k*_2_ of the E/P system decreased. This reduction can be attributed to the incorporation of flexible rubbery chains from PSR into the epoxy matrix. The introduced long-chain flexible segments dilute the concentration of reactive groups, while simultaneously increasing steric hindrance that impedes effective contact between epoxy groups and the curing agent. Consequently, both the non-catalyzed and autocatalyzed reaction rates are slowed down. Notably, the E/P/F system exhibits a lower *k*_2_ value compared to the EP and E/P systems, consistent with its reduced maximum curing rate observed in the dα/dt-α curve (Figure 4d). The elevated *k*_2_ in the EP system indicates intense autocatalytic behavior during the curing stage, which potentially leads to localized temperature spikes or thermal runaway. Such effects can induce side reactions [48], complicate resin processing and storage [49], and ultimately yield an uneven cross-linked network with compromised performance [50]. In contrast, the E/P/F system demonstrates a milder autocatalytic process, which can help mitigate side reactions and facilitate the formation of a more homogeneous network with higher cross-linking density. This is consistent with the phenomenon that the curing exothermic peak of the E/P/F system gradually broadens with the increase of FPOSS content (Figure 4b), which is attributed to the fact that FPOSS promotes the rapid curing of epoxy resins, a point that is further elaborated in the subsequent section. This acceleration leads to the increase of system viscosity and restricts the mobility of polymer chains even before entering into the self-catalytic stage, thus slowing down the overall exothermic process. As a result, a more gradual curing pathway is established, which favors the formation of a homogeneous network structure.

**Figure 5:**
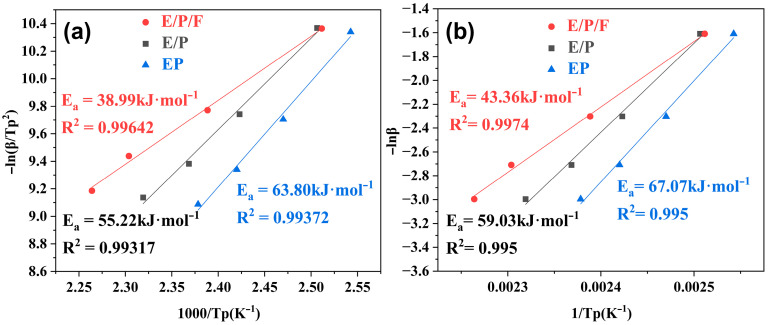
Fitting curves of the exothermic peak temperature vs. heating rate of EP, E/P and E/P/F mixtures based on the Kissinger equation (**a**) and the Ozawa equation (**b**).

Furthermore, the activation energy (*E_a_*) for the epoxy-curing agent reaction was calculated using the Kissinger equation (Equation (2)) and the Ozawa equation (Equation (3)) [51,52]:

(2) $$\ln(\frac{\beta }{T_{p}^{2}})=\ln(\frac{AR}{E_{a}})-\frac{E_{a}}{RT_{p}}$$

(3) $$\ln\beta =-5.331-1.052((\frac{E_{a}}{RT_{p}})+\ln(\frac{AE_{a}}{R})-\ln f\left(\alpha \right))$$

where *E_a_* is the activation energy (kJ/mol), *A* is the pre-exponential factor (min^−1^), R is the ideal gas constant (8.314 J/mol K), *β* is the heating rate (K/min), and *T_p_* is the peak exothermic temperature (K). Figure 5a,b present the fitted curves of -ln(*β*/*T_p_^2^*) versus 1000/*T_p_* and ln(*β*) versus 1/*T_p_*, respectively. All three epoxy systems exhibit strong linear correlations across both methods. The calculated activation energies are summarized in Table S2. Both methodologies confirm a significant reduction in *E_a_* for the E/P/F system. The reduction in activation energy is consistent with the following facts: the initial curing temperature progressively decreases with higher FPOSS content (Table S3), while the *k*_1_ value of the E/P/F system exceeds those of both EP and E/P systems (Table 2). These results demonstrate that FPOSS incorporation effectively lowers the reaction energy barrier, facilitating earlier curing initiation prior to the dominance of the autocatalytic stage. This effect is primarily attributed to the catalytic role of the hydroxyl groups in FPOSS molecules in the amine–epoxy ring-opening reaction [53,54]. Specifically, the proton in the hydroxyl group can form hydrogen bonds with the oxygen atom in the epoxy group [55], while the hydroxyl group, acting as a Lewis acid, synergistically enhances the electronegativity of the epoxy group through hydrogen bonding with the proton, thereby increasing its reactivity toward nucleophiles and promoting the formation of the epoxy–amine structure. FPOSS not only effectively catalyzes the curing reaction but also provides abundant nucleation sites due to its high specific surface area and numerous surface reactive groups, which can shorten the non-catalytic initial stage, accelerates the transition into the autocatalytic stage. Calculated values reveal that the E/P/F system possesses a comparatively lower pre-exponential factor (A), suggesting the reduced reaction rate constant relative to the other systems (Table S4). Consequently, this delays the onset of autocatalytic acceleration, thereby suppressing the rapid growth rate characteristic of the autocatalytic reaction stage during curing. In addition, the optimal curing process was determined (see S4 Section of the Supporting Information).

These kinetic results confirm that FPOSS exhibits significant advantages in regulating the curing behavior of epoxy resins. Its mechanism of action involves significantly accelerating the curing process while effectively suppressing vigorous autocatalytic reactions, enabling it to proceed in a milder and more controlled manner compared to the neat EP system, thereby facilitating the formation of a more uniform cross-linked network structure [46]. This molecular-level regulation ultimately influences the physical properties of the cured product.

### 3.4. Mechanical Properties of Epoxy Resins

**Figure 6:**
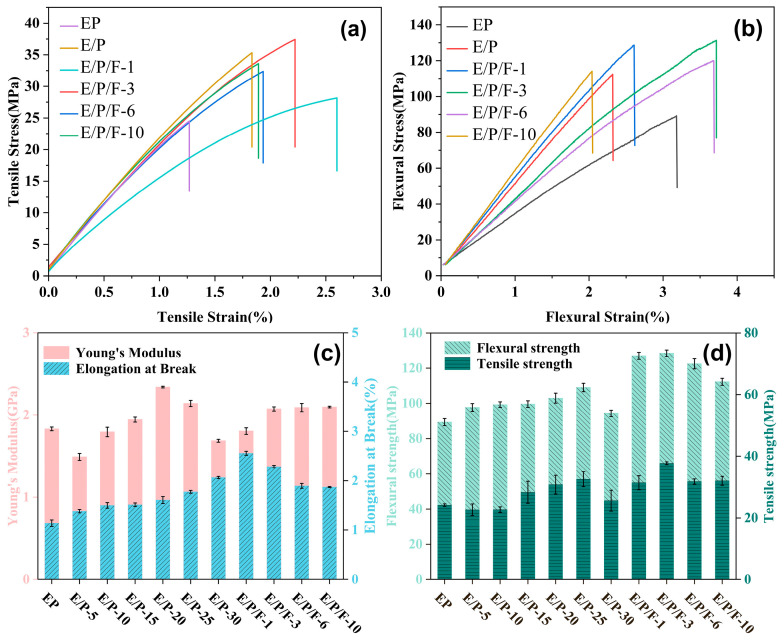
(**a**) Tensile stress–strain curves of EP, E/P and E/P/F-x resins; (**b**) Flexural stress–strain curves of EP, E/P and E/P/F-x resins; (**c**) Effect of the content of PSR and FPOSS on the Young’s modulus and elongation; (**d**) Effect of the content of PSR and FPOSS on the tensile strength and flexural strength.

Tensile and flexural tests were conducted on E/P-x and E/P/F-x nanocomposites to characterize the influence of PSR and FPOSS on the mechanical properties. The mechanical properties of the cured EP varying PSR and FPOSS content are presented in Figure 6, with corresponding detailed data provided in Table S7. It is obvious that all modified epoxy resin systems demonstrated enhanced ductility compared to pure EP. Through systematic compositional modulation, the optimal toughening component content for PSR was determined (Figures S8 and S9). When FPOSS is incorporated for synergistic modification (E/P/F series), the strength and toughness of the epoxy thermosets are further enhanced. Specifically, the E/P/F-3 sample exhibits a Young’s modulus of 2.07 GPa, tensile strength of 37.74 MPa, flexural strength of 128.48 MPa, and elongation at break of 2.07%, representing increases of 13%, 56%, 44%, and 101%, respectively, compared to the pristine EP. This performance enhancement is primarily attributed to the rigid POSS structure, which introduces additional entanglement sites via nucleophilic addition reactions and improves dispersion within the matrix [56]. Additionally, FPOSS, characterized by its small hydrodynamic volume and spherical dispersion morphology [57], effectively penetrates and uniformly distributes between the molecular chains of the resin matrix. This facilitates the formation of nanoscale crosslinking points, enabling efficient load transfer. However, a further increase in FPOSS content (e.g., in E/P/F-6 and E/P/F-10 samples) results in a slight decline in both strength and toughness. Firstly, the spatial steric hindrance effect induced by localized over-crosslinking reduces the overall crosslinking density of the system. Moreover, the agglomeration of FPOSS particles driven by strong intermolecular interactions compromises their compatibility with the resin matrix and induces the unfavorable stress concentrations [43]. 

**Figure 7:**
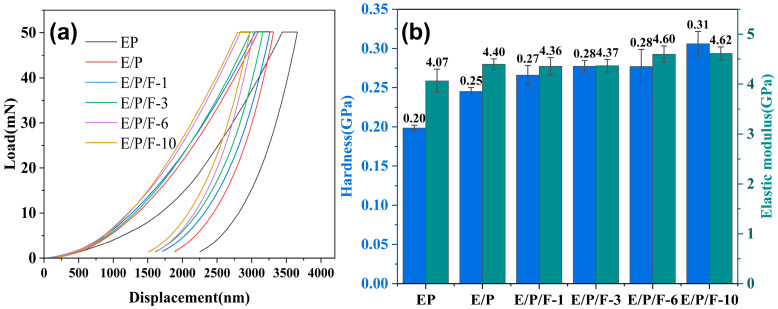
(**a**) Load–displacement curves of EP, E/P and E/P/F resins with a maximum load of 50 mN; (**b**) The hardness (H) and elastic moduli (E) of EP, E/P and E/P/F resins calculated from the obtained load–displacement curves.

To further evaluate the mechanical properties of the resin at the micrometer scale, nanoindentation techniques were employed to characterize the modulus (E) and hardness (H) [58]. Figure 7a presents typical indentation load–displacement curves for the different samples. It is observed that the peak displacement (h_max_) gradually decreases at the maximum load (50 mN) with increasing FPOSS content, while the slope of the unloading curve progressively increases. This trend demonstrates an enhancement in the stiffness and strength of this system, indicating that the E/P/F system exhibits stronger resistance to external forces. Based on the unloading segment of the load–displacement curves, the modulus and hardness values are calculated and summarized in Figure 7b. Notably, when the FPOSS content reaches 10%, the E/P/F system exhibits high E and H values of 4.62 GPa and 0.31 GPa, respectively. Considering that nanoindentation testing primarily reflects material heterogeneity rather than assessing its integral mechanical behavior, the choice of testing locations significantly influences the results. Even though excessive nanofillers induced localized agglomerates and an uneven distribution of FPOSS, which caused the enhancement in nanomechanical properties to lag behind the improvement in tensile performance, this phenomenon nevertheless precisely underscores the considerable reinforcing potential of FPOSS as a rigid nanofiller.

### 3.5. Morphology and the Toughening Mechanism of Epoxy Resins

**Figure 8:**
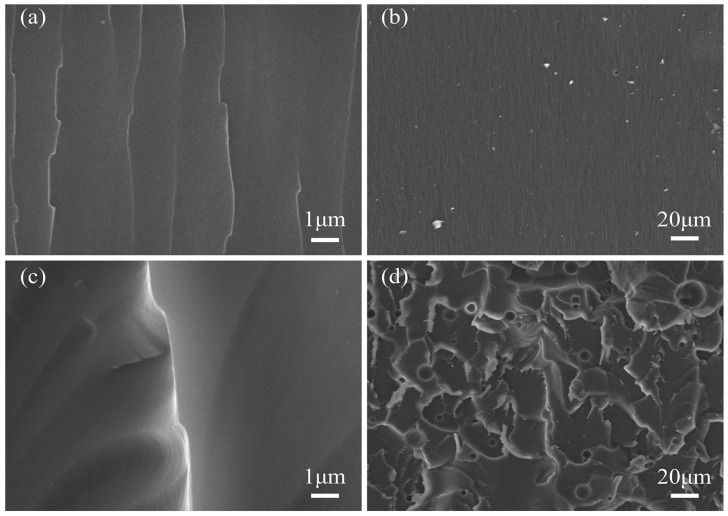
SEM images of the fracture surfaces morphology of the cured hybrids: (**a**) EP at 10,000×, (**b**) EP at 500×, (**c**) E/P-25 at 10,000×, and (**d**) E/P-25 at 500×.

**Figure 9:**
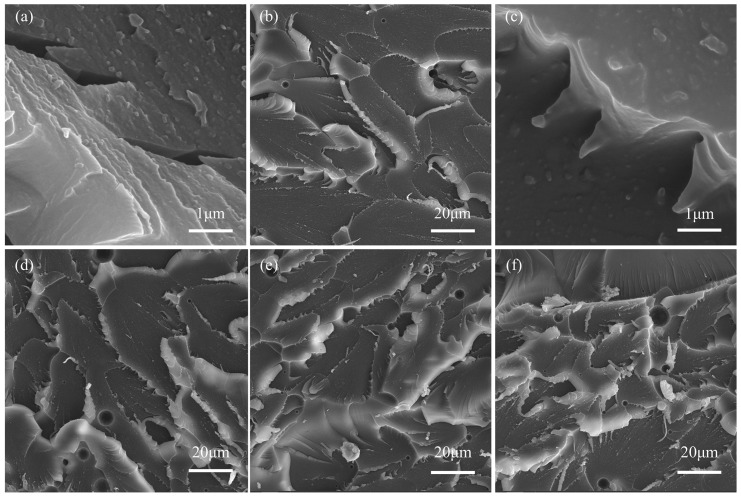
SEM images of the fracture surfaces morphology of the cured hybrids: (**a**) E/P/F-1 at 50,000×, (**b**) E/P/F-1 at 2000×, (**c**) E/P/F-3 at 50,000×, (**d**) E/P/F-3 at 2000×, (**e**) E/P/F-6 at 2000× and (**f**) E/P/F-10 at 2000×.

Figure 8 and Figure 9 show the SEM micrographs of the fracture surfaces of different samples following tensile failure. As depicted in Figure 8a,b, the fracture surface of pure EP appears smooth, exhibiting sparse, straight cracks with no significant deflection, indicating typical brittle fracture behavior. In contrast, fracture surfaces of epoxy resins modified with various toughening agents display markedly higher surface roughness, characterized by numerous wrinkles, wavy cracks accompanied by delamination, and crack path deflection, termination, and deviation from the original plane. These features collectively represent characteristic morphological signatures of ductile fracture. The fracture morphology of the PSR-toughened epoxy resin is shown in Figure 8c,d. This sample exhibits more extensive plastic shear deformation within the matrix. Additionally, distinct cavitation voids are evident on the fracture surface, originating from the debonding and pull-out of rubber particles from the epoxy matrix [53]. Significantly different fracture morphologies are observed for the E/P/F nanocomposites (Figure 9). Surface roughness increases progressively with POSS content. Beyond matrix plastic deformation, distinct bright particles are visible on the fracture cross-section. These particles formed as the resin matrix undergoes plastic deformation following filler particle debonding. This mechanism is considered the primary toughening mechanism for this nanocomposite system.

Specifically, at low POSS content (e.g., E/P/F-1 and E/P/F-3; Figure 9a–d), no significant large-scale agglomeration was observed, indicating good compatibility between POSS and the EP matrix. This was further confirmed by EDS mapping showing uniform distribution of silicon elements (Figure S10). Such favorable compatibility and dispersion positively contribute to the mechanical properties of the cured composite [56], aligning with mechanical testing results. The fracture surfaces exhibited lamellar stacking patterns, stress-whitened zones, increased crack path deviation from the original plane, pronounced warping, and a marked rise in nanoparticle debonding and matrix shear deformation. In contrast, the morphology of the high-POSS-content sample (E/P/F-10; Figure 9f) differed substantially. Here, FPOSS particles formed micrometer-scale agglomerates, reducing particle debonding and yielding irregularly arranged crack patterns. Critically, the agglomerate size exceeded the dimensions of internal crack voids, physically preventing nanoparticle infiltration into crack interiors. This obstruction inhibited crack propagation, ultimately diminishing toughening efficacy.

The rigid inorganic cage-like hollow core of POSS serves as a stress concentration point that bears external loads. When cracks initiate and propagate through the matrix, POSS particles act as impediments, inducing crack deflection and bifurcation via their eight-arm architecture [59]. This process delays or arrests crack advancement, establishing a “bridging constraint effect” [60]. Consequently, crack tips fail to penetrate the rigid POSS structures with strong interfacial adhesion, causing crazes to deflect, warp, or terminate near the particles [9]. Stress concentration at the crack tip transfers to the toughening phase, triggering bulging and secondary crack formation. These secondary cracks may subsequently coalesce after circumventing the toughening phase, generating characteristic fracture steps. This phenomenon demonstrates the toughening phase’s pinning effect, aligning with the “crazing pinning” toughening mechanism. The associated crack deflection and warping further promote local shear band formation [61]. Upon stress transfer to POSS particles, particle debonding or cavitation occurs, initiating plastic void growth. This leads to the development of a ductile shear zone at the crack–cavity interface, which subsequently induces scale-like plastic deformation in the resin matrix. The shear zone blunts the crack tip, reducing local stress concentration while promoting warping (Figure 9) [62]. Collectively, this process inhibits crack propagation and dissipates mechanical energy [61], ultimately achieving a “crazing-shear band” synergistic toughening mechanism [62]. 

**Figure 10:**
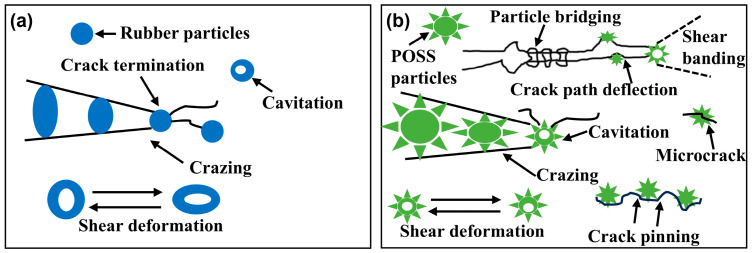
Schematic representation of the synergistic toughening mechanism of E/P/F ternary composites: (**a**) Role of rubber particles; (**b**) Role of POSS particles.

Meanwhile, POSS forms covalent bonds with the resin matrix through cross-linking reactions, enabling effective load transfer across the cross-linked network to the flexible Si-O-Si chain segments [57]. These segments, along with the introduced flexible rubber molecular chains, preferentially undergo deformation rather than fracture. This behavior enhances the resin’s capacity for plastic deformation under applied force. Consequently, this mechanism facilitates stress dissipation, suppresses crack initiation and propagation, and ultimately enhances toughness [63]. In the synergistic toughening of epoxy resin by POSS combined with rubber segments, the rubber phase absorbs energy via shear deformation and cavitation. Concurrently, plastic deformation initiated by debonding of POSS particles induces crazes and shear band formation (Figure 10). These processes promote further stress dispersion within the three-dimensional resin matrix. As a result, the fracture surface morphology exhibits increased roughness, correlating with significantly enhanced resin toughness. Furthermore, DSC analysis indicates that FPOSS moderates the curing process of the epoxy resin. This gradual curing process reduces internal stress generation during crack formation. In summary, the microstructural characteristics observed on the fracture surfaces provide direct morphological evidence for the synergistic enhancement of both strength and toughness in the E/P/F system.

### 3.6. Thermal Properties of Epoxy Resins

**Figure 11:**
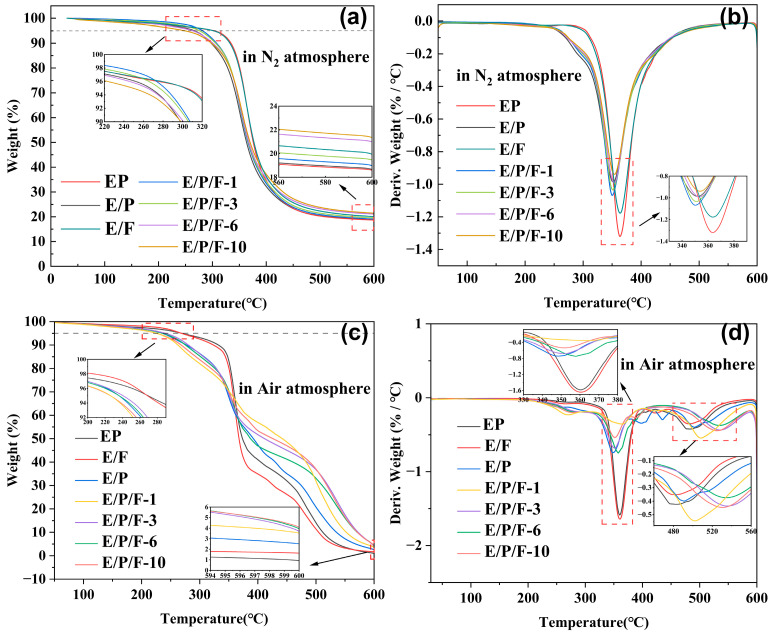
TGA (**a**) and DTG (**b**) curves of EP, E/P, E/F and E/P/F-x resins under N_2_ atmosphere with a heating rate of 10 °C·min^−1^; TGA (**c**) and DTG (**d**) curves of EP, E/P, E/F and E/P/F-x resins under air atmosphere with a heating rate of 10 °C·min^−1^.

The thermal stability of the samples was evaluated using TGA nitrogen and air atmospheres, respectively. Key parameters, including the initial decomposition temperature (T_5%_), the maximum degradation temperature (T_max_), and the char yield at 600 °C, were determined. The corresponding TGA and DTG curves are presented in Figure 11, with specific data summarized in Table S8. Notably, the char yield at 600 °C increased steadily with increasing POSS content. Compared to the pure epoxy cured material, the introduction of FPOSS significantly reduced the maximum thermal weight loss rate, indicating an effective improvement in the thermal degradation stability of the system. This enhancement is attributed to the fact that the inorganic cage-like framework of POSS undergoing thermal oxidation can generate stable silicon oxides via condensation reaction. Additionally, the carbon layer and the silicon oxide layer synergistically exert a physical barrier effect, which effectively shields the underlying substrate from high-temperature exposure and flame erosion while simultaneously suppressing the release of volatile thermal degradation products [64]. As shown in Figure 11, the thermal degradation of the cured material under an air atmosphere occurs in two distinct stages. The first stage (310–380 °C) primarily involves the cleavage of chemical bonds (C–H, C–O, and C–C), leading to degradation of the polymer crosslinking network. The second stage (approximately 460–560 °C) is dominated by the oxidation of unstable carbon residues. Crucially, oxygen promotes the thermal decomposition of both siloxane and carbon chains, accelerating siloxane layer formation. Consequently, the char residue of E/P/F-10 reached 4.41 times that of the pure epoxy cured material, representing a significant improvement compared to the 1.15-fold increase observed under nitrogen atmosphere. Furthermore, the maximum thermal weight loss rate temperature (*T_p2_*) in the second stage of E/P/F-3 was significantly higher than that of the pure epoxy cured material (increased by approximately 50 °C). This enhancement stems from the complete formation of a protective carbon layer, which effectively delays further decomposition of the polymer main chain, thereby significantly improving the material’s thermal stability [65]. Figure S13 presents the microstructure of residues obtained from cured pure EP and the E/P/F-6 composite after holding at 600 °C for 30 min in a muffle furnace. The residue of pure EP shows numerous open pores (Figure S13a,b), which can serve as channels for combustible gases to penetrate the interior and prevent the formation of an effective thermal and oxygen barrier to shield the underlying matrix. In contrast, the carbonaceous residue of E/P/F-6 exhibits a dense and compact char layer without visible surface pores (Figure S13c–f). This coherent char acts as an efficient physical barrier at elevated temperatures, effectively inhibiting oxygen diffusion and heat transfer, thereby offering enhanced protection to the epoxy matrix.

T_g_ measurements directly indicate interactions between polymer chains and POSS units. As shown in Figure S11, all samples exhibit a single T_g_ in their DSC curves, confirming homogeneous dispersion of POSS within the epoxy matrix and the absence of macroscopic phase separation. Typically, incorporating POSS as side-chain units reduces T_g_ in epoxy systems [66,67]. However, this study revealed that adding 3 wt.% FPOSS increased T_g_ by 4.12 °C. More significantly, in systems where flexible rubber chains substantially reduced T_g_ (E/P system), FPOSS addition markedly mitigated this decrease—with the E/P/F-3 sample exhibiting a 16.82 °C T_g_ increase (Table S8). This anomaly primarily stemmed from our structural design strategy: (1) Chemically bonded rigid POSS cages and furan rings restrict segmental mobility of adjacent polymer chains [33]; (2) Multiple crosslinking sites elevate network crosslink density, forcing molecular segments to overcome higher activation energy barriers during motion. Consequently, these synergistic effects drove the observed macroscopic T_g_ enhancement.

As the POSS content increases further, T_g_ exhibits a decreasing trend. While previous studies have attributed such T_g_ reductions to incomplete curing reactions induced by POSS introduction [34], FTIR analysis of the E/P/F system (Figure S12) reveals the complete disappearance of the epoxy ring characteristic absorption peak at 910 cm^−1^ after curing. This observation confirms the full completion of the epoxy curing reaction. For the present system, the T_g_ reduction can be attributed to the fact that the bulky cage-like structure of POSS promotes aggregation, increasing intermolecular distances, weakening intermolecular forces, and consequently augmenting the system’s free volume. This interpretation aligns with the corresponding morphological observations (Figure 9f). It is pertinent to emphasize that purely physically blended nanofillers typically exert minimal influence on T_g_, as they do not significantly alter the molecular chain mobility of the matrix resin [68]. However, the significant T_g_ decrease observed in the E/P/F-10 system demonstrates that FPOSS introduction involves more than mere physical blending. Instead, FPOSS effectively participates in the construction of the epoxy network structure through chemical bonding.

## 4. Conclusions

In this work, a furan-ring-grafted POSS was synthesized and, together with liquid polysulfide rubber, utilized as the multi-component toughening agent to simultaneously enhance the strength and toughness of EP. Microscopic morphology analysis unveils a unique “hard–soft” biphasic synergistic toughening mechanism: the polysulfide rubber phase dissipates energy through shear deformation and cavitation, while simultaneously, the dispersed rigid POSS particles undergo stress-induced delamination. This delamination induces plastic deformation of the matrix and promotes the formation of silver streaks and shear bands, thereby substantially enhancing the material’s toughness. DSC analysis reveals that FPOSS effectively suppresses the vigorous autocatalytic reaction of the epoxy system, enabling the epoxy groups in the E/P/F system to cure in a milder manner. Notably, the incorporation of 3 phr FPOSS and 25 phr PSR resulted in a modified epoxy resin exhibiting a 13% increase in Young’s modulus, a 56% enhancement in tensile strength, and a 101% improvement in elongation at break. This significant mechanical reinforcement is attributed to the synergistic effect of flexible rubber chains and rigid POSS particles, which form additional entanglement sites and achieve superior dispersion through nucleophilic addition. Furthermore, the addition of FPOSS effectively mitigates the depression in T_g_ caused by the rubber phase, yielding a 16.82 °C increase in T_g_. Under a 600 °C air atmosphere, the residual char yield of the modified epoxy resin was 4.41 times greater than that of the pure epoxy resin, indicating that FPOSS significantly promotes the formation of a protective char layer and confers enhanced thermal degradation stability. The modification strategy proposed herein successfully achieves an optimized balance between strength, toughness, and high-temperature stability in epoxy resins, providing valuable insights for the preparation of high-performance epoxy resins and their applications in fields such as composite materials.

## Figures and Tables

**Table 1:** Surface element content of EP-POSS and FPOSS.

Samples	C1s (mol%)	O1s (mol%)	Si2p (mol%)	N1s (mol%)
EP-POSS	57.41	33.46	9.13	/
FPOSS	62.92	25.47	7.35	4.26

**Table 2:** Kinetic parameters of the Kamal–Sourour model for EP, E/P and E/P/F.

Samples	*k*_1_ (min^−1^)	*k*_2_ (min^−1^)	*m*	*n*	*m* + *n*	R^2^
EP	0.0021	0.6599	0.7003	0.8801	1.5804	0.9978
E/P	0.0001	0.5927	0.7427	0.7839	1.5266	0.9964
E/P/F	0.0040	0.5117	0.6276	0.6884	1.3160	0.9987

## Data Availability

The raw data supporting the conclusions of this article will be made available by the authors on request.

## Supplementary Materials

The following supporting information can be downloaded at: https://www.mdpi.com/article/10.3390/polym18020184/s1, Figure S1: The chemical structures of AFG-90 (I), KH-560 (II) and PSR (III).; Table S1: Formulation of synthesizing E/P-x and E/P/F-x. Figure S2: High-resolution mass spectrum (HRMS) of EP-POSS. Figure S3: High-resolution XPS spectra of C1s (a) and O1s (b) of EP-POSS; Figure S4: (a) FT-IR spectrum of EP-POSS, FA and FPOSS; (b) ^1^H NMR spectrum of FPOSS; (c) FT-IR spectrum of EP and the uncured hybrids (EP-PSR and EP-PSR-FPOSS); (d) ^13^C NMR spectrum of FPOSS. Figure S5: TGA curve of FPOSS under N_2_ atmosphere with a heating rate of 10 °C/min. Figure S6: Distribution of FPOSS particle size in EP-PSR system. Table S2: The apparent activation energy and correlation coefficient of curing reaction of EP, E/P and E/P/F. Table S3: The parameters of curing reaction of EP, E/P, E/F and E/P/F-x. Table S4: Index factor of curing reaction of EP, E/P and E/P/F. Figure S7: DSC thermograms of EP (a), E/P(b) and E/P/F(c) mixtures at different heating rates; Fitting curves of characteristic temperature of EP(d), E/P(e) and E/P/F(f) mixtures with different heating rates. Table S5: The parameters of curing reaction of EP, E/P and E/P/F at different heating rates. Table S6: Curing process of EP, E/P and E/P/F. Figure S8: Tensile stress–strain curves of EP and E/P-x resins. Figure S9: Flexural stress–strain curves of EP and E/P-x resins. Table S7: The specific parameters of mechanical properties of E/P-x and E/P/F-x. Figure S10: EDS mapping (a–c) of the E/P/F-3 surface. Table S8: Thermal properties of E/P-x and E/P/F-x. Figure S11: DSC thermograms of EP, E/P, E/F and E/P/F-x hybrids. Figure S12: FT-IR spectrum of EP, the uncured and cured hybrids (E/P/F-10). Figure S13. SEM images of the residues: (a) EP at 50×, (b) EP at 200×, (c) the exterior of E/P/F-6 at 50×, (d) the exterior of E/P/F-6 at 200×, (e) the interior of E/P/F-6 at 50×, and (f) the interior of E/P/F-6 at 200×.
