# Supplementary material for: Enhanced Mechanical and Thermal Properties of Epoxy Resins Through Hard–Soft Biphasic Synergistic Toughening with Modified POSS/Polysulfide Rubber
Source: Polymers (Basel). 2026 Jan 9;18(2):184. doi: 10.3390/polym18020184 (PMC12845993; doi:10.3390/polym18020184)
Supplement: Supplementary file 1 [file polymers-18-00184-s001.zip › polymers-4062222-SI.pdf]

Supporting Information

Enhanced Mechanical and Thermal Properties of Epoxy Resins Through Hard–Soft Biphasic Synergistic Toughening with Modified POSS/Polysulfide Rubber

Xi Yuan [1], Zhineng Tan [1], Shengwen Liu [2], Hang Luo [2], Zhuo Chen [2,*] and Dou Zhang [2,*]

[1] College of Chemistry and Chemical Engineering, Central South University, Changsha 410083, China; xiyuan@csu.edu.cn (X.Y.); 232311082@csu.edu.cn (Z.T.)
[2] State Key Laboratory of Powder Metallurgy, Central South University, Changsha 410083, China; shengwenliu@csu.edu.cn (S.L.); hangluo@csu.edu.cn (H.L.)
* Correspondence: cz2009@csu.edu.cn (Z.C.); dzhang@csu.edu.cn (D.Z.)

Contents

S1. Materials and Methods........2
Figure S1. The chemical structures of AFG-90 (I), KH-560 (II) and PSR (III). 2
Table S1. Formulation of synthesizing E/P-x and E/P/F-x. 2
S2. Synthesis and Characterization of EP-POSS........2
Figure S2. High-resolution mass spectrum (HRMS) of EP-POSS........3
Figure S3. High-resolution XPS spectra of C1s (a) and O1s (b) of EP-POSS. 3
S3. Characterization of FPOSS 3
Figure S4. (a) FT-IR spectrum of EP-POSS, FA and FPOSS; (b) ^{1}H NMR spectrum of FPOSS; (c) FT-IR spectrum of EP and the uncured hybrids (EP-PSR and EP-PSR-FPOSS); (d) ^{13}C NMR spectrum of FPOSS. 5
Figure S5. TGA curve of FPOSS under N_2 atmosphere with a heating rate of 10 °C/min. 6
Figure S6. Distribution of FPOSS particle size in EP-PSR system........6
S4. Curing Behaviors and Mechanism........6
Table S2. The apparent activation energy and correlation coefficient of curing reaction of EP, E/P and E/P/F. 6
Table S3. The parameters of curing reaction of EP, E/P, E/F and E/P/F-x......6
Table S4. Index factor of curing reaction of EP, E/P and E/P/F. 7
Figure S7. DSC thermograms of EP (a), E/P (b) and E/P/F (c) mixtures at different heating rates; Fitting curves of characteristic temperature of EP(d), E/P(e) and E/P/F(f) mixtures with different heating rates. 8
Table S5. The parameters of curing reaction of EP, E/P and E/P/F at different heating rates. 8
Table S6. Curing process of EP, E/P and E/P/F. 8

S5. Mechanical Properties of Epoxy Resins 9
Figure S8. Tensile stress–strain curves of EP and E/P-x resins. 9
Figure S9. Flexural stress–strain curves of EP and E/P-x resins. 10
Table S7. The specific parameters of mechanical properties of E/P-x and E/P/F-x. 10
S6. Morphology and the Toughening Mechanism of Epoxy Resins 10
Figure S10. EDS mapping (a-c) of the E/P/F-3 surface. 10
S7. Thermal Properties of Epoxy Resins 10
Table S8. Thermal properties of E/P-x and E/P/F-x. 11
Figure S11. DSC thermograms of EP, E/P, E/F and E/P/F-x hybrids.. 11
Figure S12. FT-IR spectrum of EP, the uncured and cured hybrids (E/P/F-10) 12
Figure S13. SEM images of the residues: (a) EP at 50×, (b) EP at 200×, (c) the exterior of E/P/F-6 at 50×, (d) the exterior of E/P/F-6 at 200×, (e) the interior of E/P/F-6 at 50×, and (f) the interior of E/P/F-6 at 200×..12

S1. Materials and Methods

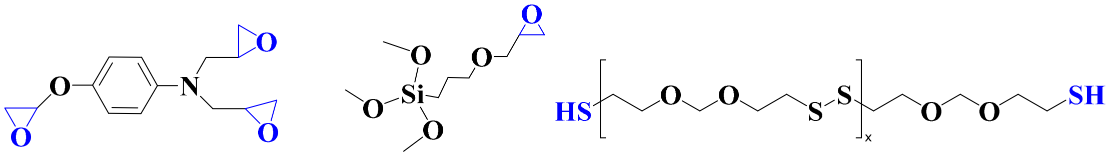

(I) **(II)** **(III)**

Figure S1. The chemical structures of AFG-90 (I), KH-560 (II) and PSR (III).

Table S1. Formulation of synthesizing E/P-x and E/P/F-x.

Samples	AFG-90 (wt.%)	PSR (wt.%)	FPOSS (wt.%)	CA-15 (wt.%)
Neat EP	100	/	/	50
E/P-5	100	5	/	50
E/P-10	100	10	/	50
E/P-15	100	15	/	50
E/P-20	100	20	/	50
E/P-25	100	25	/	50
E/P-30	100	30	/	50
E/F	100	/	3	50
E/P/F-1	100	25	1	50
E/P/F-3	100	25	3	50
E/P/F-6	100	25	6	50
E/P/F-10	100	25	10	50

S2. Synthesis and Characterization of EP-POSS

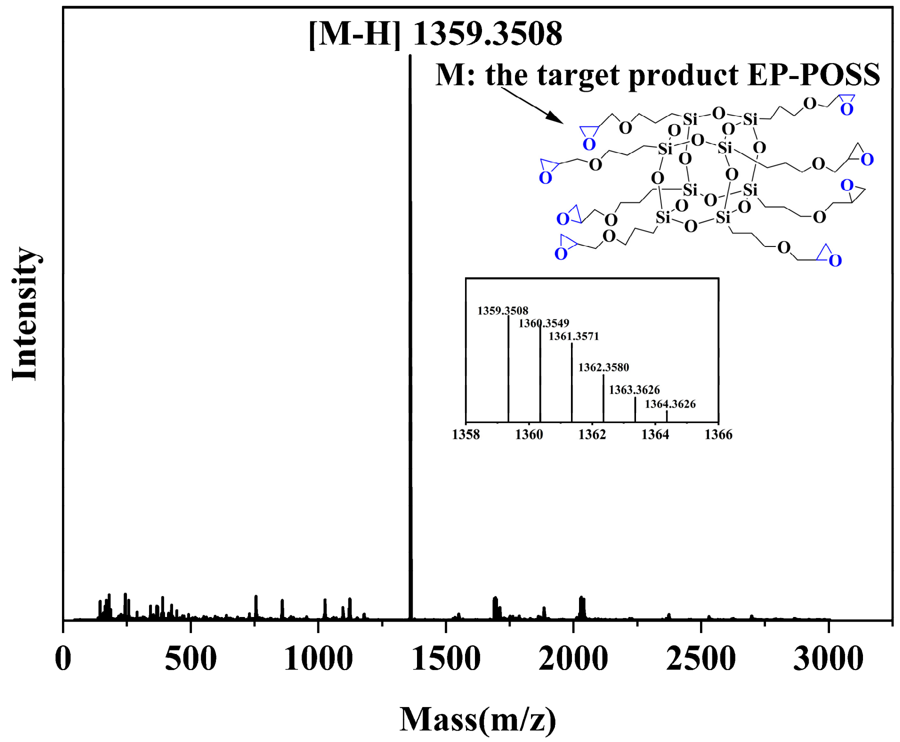

Figure S2. High-resolution mass spectrum (HRMS) of EP-POSS.

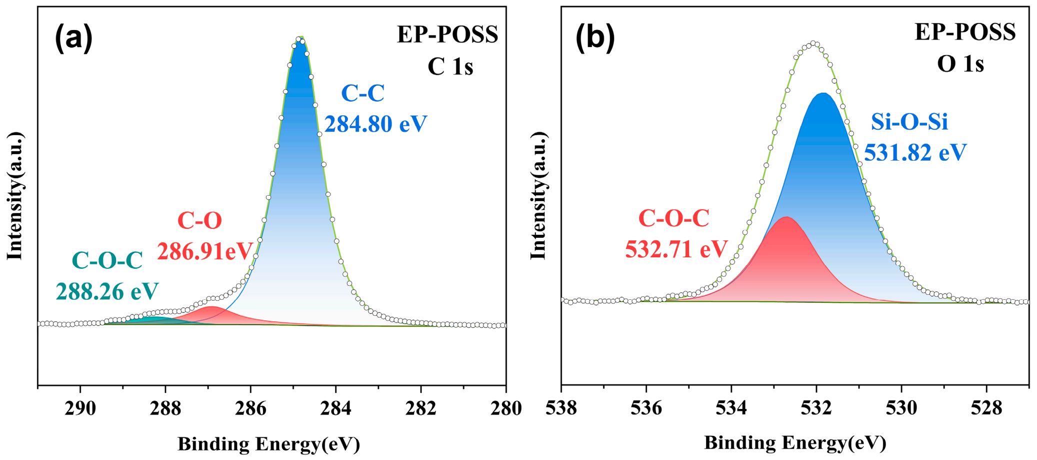

Figure S3. High-resolution XPS spectra of C1s (a) and O1s (b) of EP-POSS.

S3. Characterization of FPOSS

Figure S4a presents the FTIR spectra of EP-POSS, FA, and FPOSS. Comparative analysis revealed the disappearance of the characteristic epoxy group absorption peak at 910 cm^{-1} (EP-POSS) and the doublet corresponding to the primary amino group (-NH_2) at 3290 cm^{-1} and 3372 cm^{-1} (FA) in the FPOSS spectrum. Simultaneously, the N-H bending vibration peak at 1603 cm^{-1} shifted to lower wavenumbers, and the characteristic peak of the newly formed secondary amino group (-NH-) overlapped with

the O-H stretching vibration, which resulted in a new broad peak centered at 3460 cm^{-1}. These spectral changes confirmed that the amino group underwent a ring-opening reaction with the epoxy group, with no residual free primary amino groups present in the reaction system. Notably, the characteristic Si-O-Si absorption bands at 1074 cm^{-1} and 1110 cm^{-1} in the FPOSS spectrum exhibited significant broadening. This observation further supported the successful grafting of FA onto the EP-POSS molecular structure.

As shown in Figure S4b, the characteristic signals corresponding to the furan ring protons were observed at 7.47 ppm, 6.31 ppm, and 6.14 ppm. The proton signal assigned to the hydroxyl group formed by the reaction between the amino group and the epoxy group appeared at 2.33 ppm, while the signal from the newly formed secondary amino group was detected at 3.00 ppm. The integral ratio of these two proton signals is approximately 1:1. For further quantification, characteristic peaks corresponding to the furan ring protons (δ = 6.14 ppm) of FPOSS and the Si–CH_2– protons (δ = 0.54 ppm) of the POSS cage backbone were selected for integration. Based on their area ratio and the number of corresponding protons, it was calculated that an average of approximately eight furan groups is grafted onto each POSS cage. This result indicates that FPOSS possesses a well-defined and quantifiable furan functionality, thereby providing sufficient reactive sites for subsequent cross-linking reactions. In the ^{13}C NMR spectrum of FPOSS (Figure S4d), characteristic signals for the furan ring carbon atoms were observed at 156.63 ppm, 141.60 ppm, 110.18 ppm, and 105.05 ppm. Additionally, characteristic signals of the alkyl linkages within the POSS skeleton were present at 29.70 ppm and 18.45 ppm. Collectively, the ^{1}H NMR and ^{13}C NMR spectral data confirm the structure of the target ring-opened adduct (FPOSS).

Through a pre-curing process, flexible rubber segments and low-content FPOSS were grafted onto the main chain of EP. FT-IR analysis of samples E/P-25 and E/P/F-3 (Figure S4c) revealed a significant increase in the intensity of the O-H characteristic peak at 3490 cm^{-1} upon incorporation of polysulfide rubber (PSR) and FPOSS. This is attributed to reactions between the thiol groups (-SH) in PSR, the secondary amino groups (-NH-), and hydroxyl groups (-OH) in FPOSS with the epoxy groups.

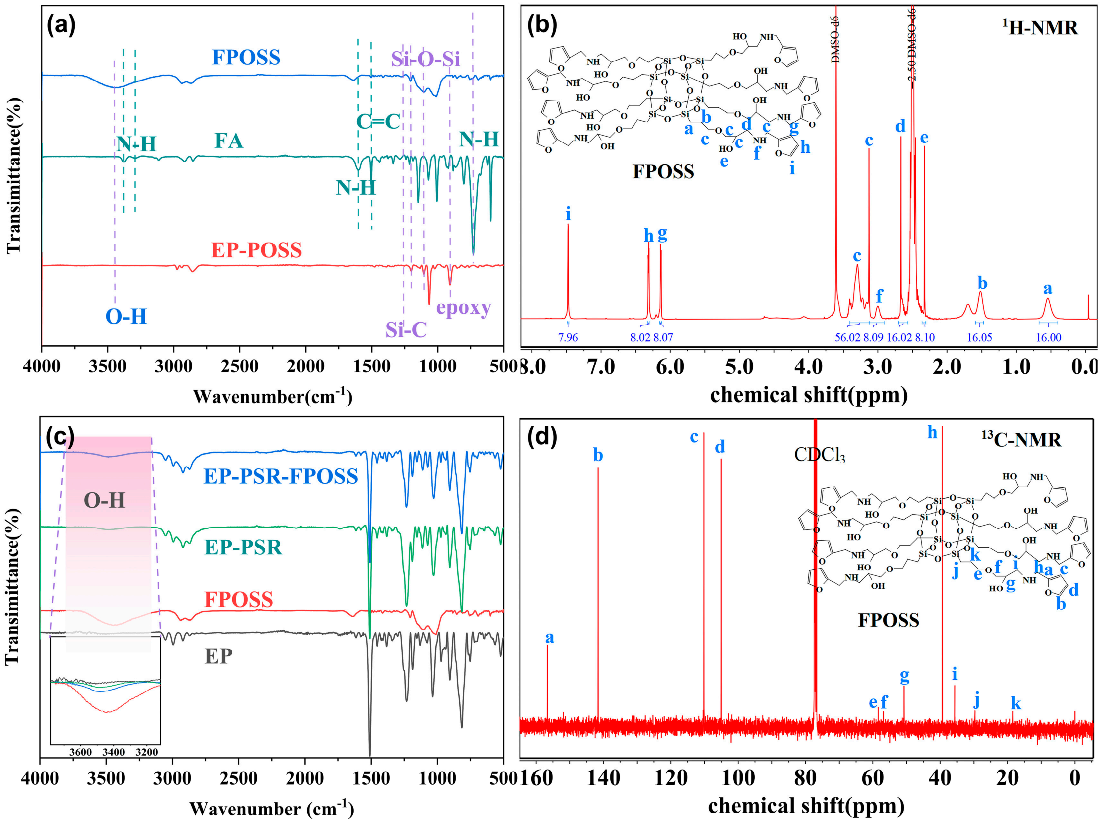

Figure S4. (a) FT-IR spectrum of EP-POSS, FA and FPOSS; (b) ^{1}H NMR spectrum of FPOSS; (c) FT-IR spectrum of EP and the uncured hybrids (EP-PSR and EP-PSR-FPOSS); (d) ^{13}C NMR spectrum of FPOSS.

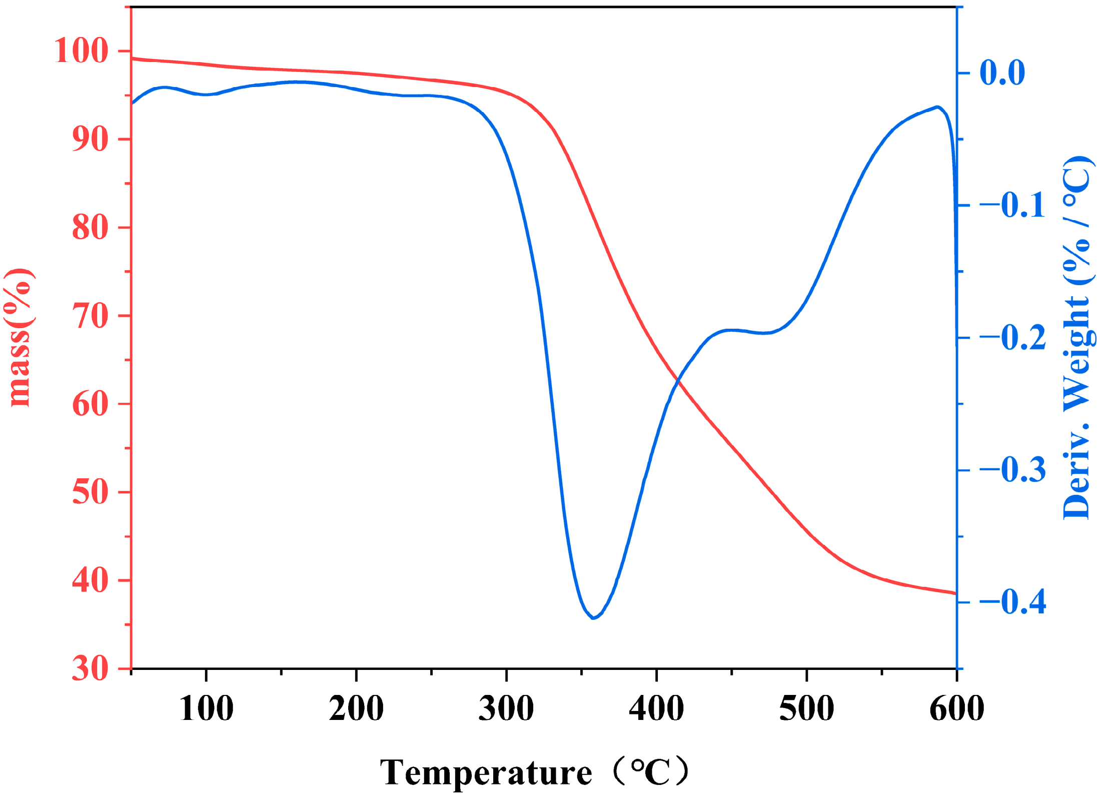

Figure S5. TGA curve of FPOSS under N_2 atmosphere with a heating rate of 10 °C/min.

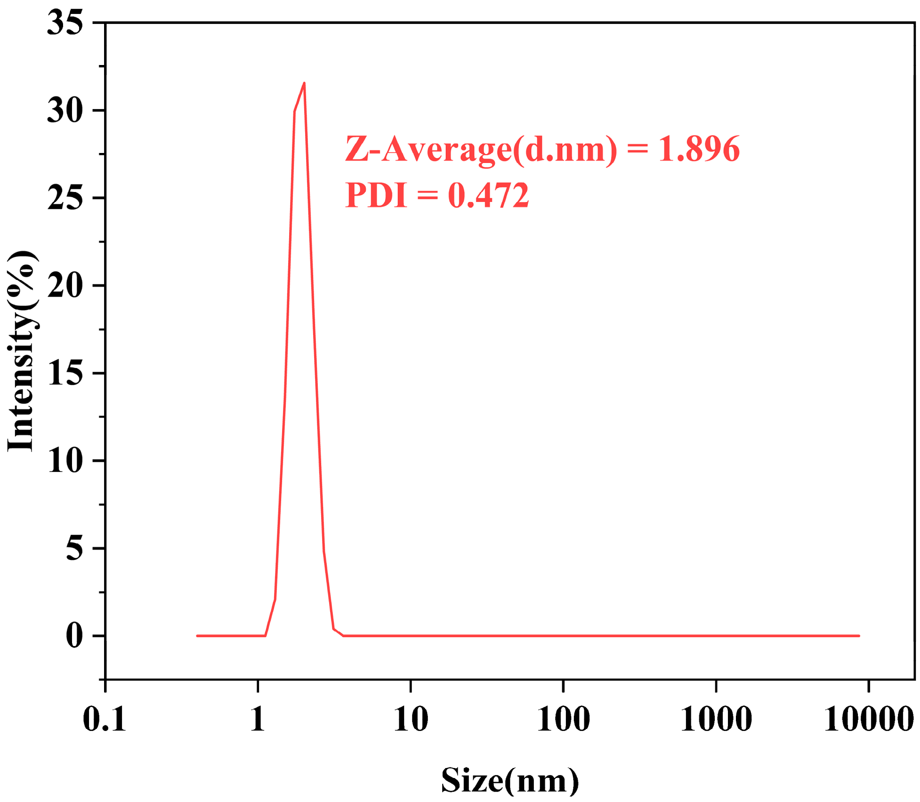

Figure S6. Distribution of FPOSS particle size in EP-PSR system.

S4. Curing Behaviors and Mechanism

Table S2. The apparent activation energy and correlation coefficient of curing reaction of EP, E/P and E/P/F.

Samples	E_a (kJ/mol)		Correlation coefficient R^2	
	Kissinger	Ozawa	Kissinger	Ozawa
EP	63.80	67.07	0.99370	0.9950
E/P	55.22	59.03	0.9932	0.9950
E/P/F	38.99	43.36	0.9964	0.9974

Table S3. The parameters of curing reaction of EP, E/P, E/F and E/P/F-x.

Samples	T_i (℃)	T_p (℃)	T_f (℃)	ΔH (J/g)
EP	102.30	130.08	168.25	46.87
E/P	96.71	138.54	173.06	59.48

E/F	92.96	133.58	169.06	50.33
E/P/F-1	91.50	137.86	172.79	36.42
E/P/F-3	90.64	141.49	173.82	36.40
E/P/F-6	88.53	139.35	173.01	36.44
E/P/F-10	87.00	140.76	174.17	36.51

Table S4. Index factor of curing reaction of EP, E/P and E/P/F.

**Samples**	**β (K/min)**	**T_p (K)**	**A (s^{-1})**	**av-A (s^{-1})**
EP	5	393.31	7.37×10^7	7.63×10^7
	10	404.86	7.98×10^7	
	15	413.22	7.83×10^7	
	20	420.48	7.32×10^7	
E/P	5	398.18	3.68×10^6	2.81×10^6
	10	418.68	2.94×10^6	
	15	434.07	2.34×10^6	
	20	441.69	2.31×10^6	
E/P/F	5	398.97	1.88×10^4	2.39×10^4
	10	412.73	2.37×10^4	
	15	422.23	2.63×10^4	
	20	431.20	2.67×10^4	

As the heating rate (β) increased, the exothermic peaks of all resin systems shifted toward higher temperatures, and the peaks became sharper (Figure S7a-c). This phenomenon was attributed to the thermal response lag of the instrument. Additionally, the three characteristic curing temperatures—initial temperature (T_i), peak temperature (T_d), and final temperature (T_f)—for all epoxy systems were plotted against the heating rate β (Figure S7d-f, Table S5) and subjected to linear fitting. The isothermal curing temperature (i.e., the minimum temperature required for cross-linking and curing) was determined by extrapolating β to 0 K/min and solving the fitted equations. This yielded key curing process parameters: gelation temperature (T_{gel}), curing temperature (T_{cur}), and post-curing temperature (T_{tre}). The corresponding curing rate equations, characteristic temperatures, and curing processes for the three epoxy systems are summarized in Table S6.

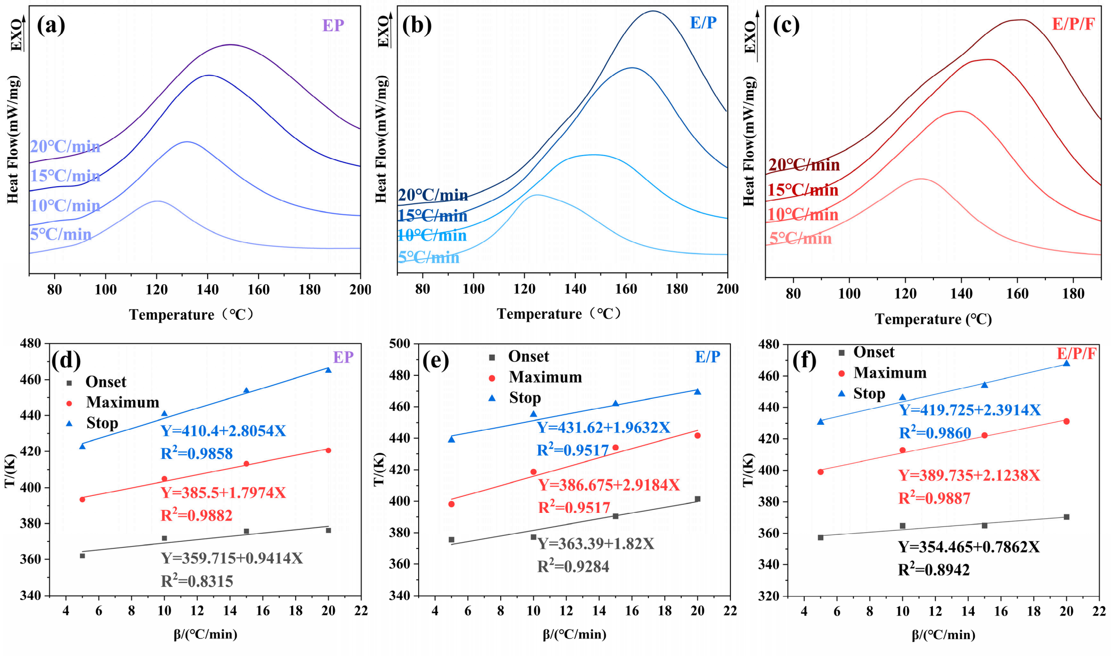

Figure S7. DSC thermograms of EP (a), E/P (b) and E/P/F (c) mixtures at different heating rates; Fitting curves of characteristic temperature of EP(d), E/P(e) and E/P/F(f) mixtures with different heating rates.

Table S5. The parameters of curing reaction of EP, E/P and E/P/F at different heating rates.

Samples	β (K/min)	T_i (K)	T_p (K)	T_f (K)
EP	5	361.96	393.31	422.42
	10	371.80	404.86	440.77
	15	375.88	413.22	453.87
	20	376.29	420.48	464.81
E/P	5	375.55	398.18	438.73
	10	377.16	418.68	454.94
	15	390.37	434.07	461.81
	20	401.48	441.69	469.16
E/P/F	5	357.21	398.97	430.53
	10	364.77	412.73	446.15
	15	364.93	422.23	454.03
	20	370.26	431.20	467.76

Table S6. Curing process of EP, E/P and E/P/F.

Samples	T_{gel} (K)	T_{cur} (K)	T_{tre} (K)	Curing rate	Curing process
EP	359.75	385.55	410.40	dα/dt = (0.0021+0.6599α^0.7003) (1-α) ^0.8801	90°C/2h+110°C/2 h+140°C/2h
E/P	363.35	386.65	431.65	dα/dt = (0.0040+0.5117α^0.6276) (1-α) ^0.6884	90°C/2h+110°C/2 h+160°C/2h

E/P/F	354.45	389.75	419.75	dα/dt = (0.0001+0.5927α^0.7427) (1-α) ^0.7839	80°C/2h+120°C/2 h+150°C/2h

S5. Mechanical Properties of Epoxy Resins

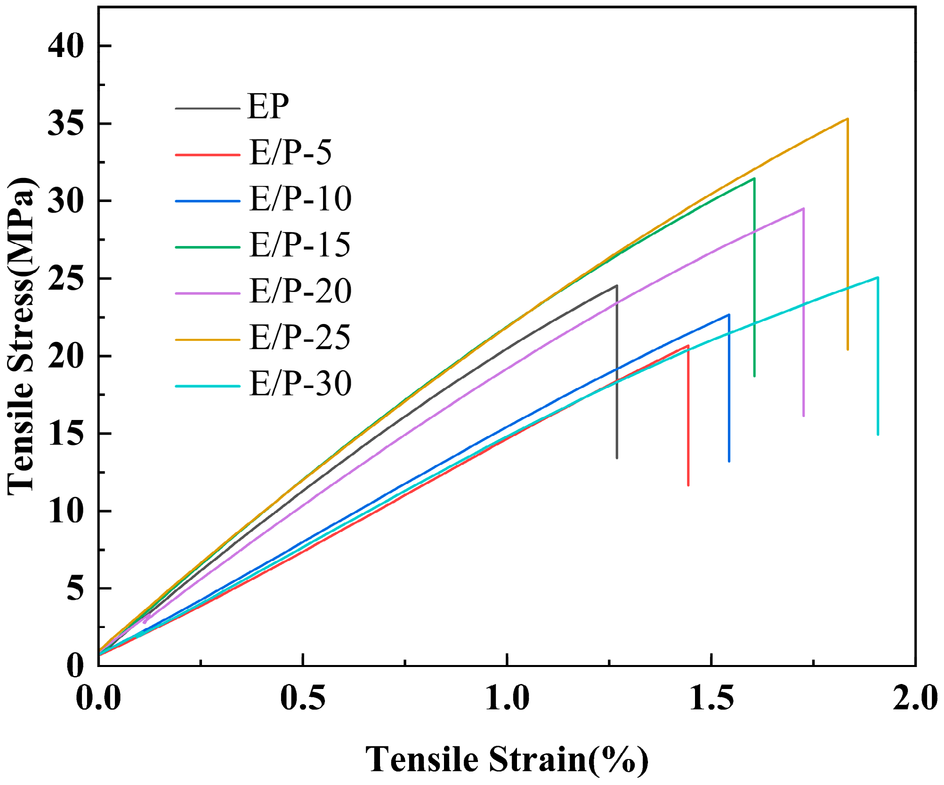

Figure S8. Tensile stress–strain curves of EP and E/P-x resins.

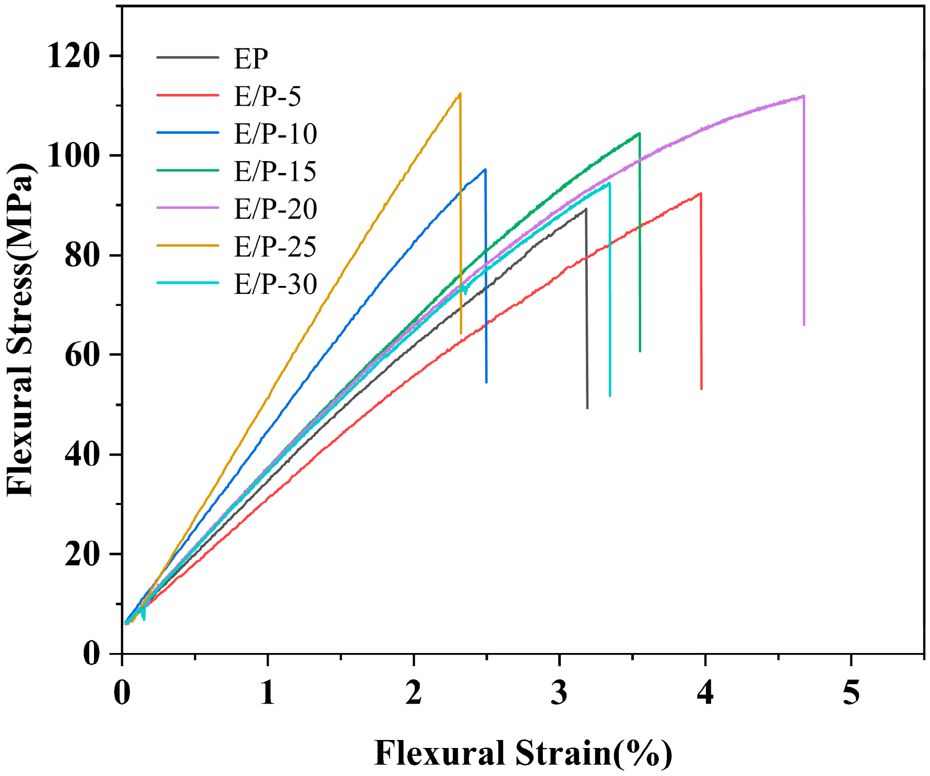

Figure S9. Flexural stress–strain curves of EP and E/P-x resins.

Table S7. The specific parameters of mechanical properties of E/P-x and E/P/F-x.

Samples	Tensile stength (MPa)	Young's modulus (GPa)	Elongation (%)	Flexural stength (MPa)
EP	24.14 ± 0.41	1.83 ± 0.02	1.14 ± 0.07	89.36 ± 2.01
E/P-5	22.63 ± 1.89	1.49 ± 0.04	1.38 ± 0.03	97.72 ± 2.18
E/P-10	22.68 ± 0.90	1.79 ± 0.06	1.50 ± 0.06	99.27 ± 1.62
E/P-15	28.30 ± 3.56	1.94 ± 0.03	1.52 ± 0.03	99.63 ± 1.85
E/P-20	30.78 ± 3.02	2.34 ± 0.01	1.61 ± 0.07	103.00 ± 2.81
E/P-25	32.53 ± 2.43	2.14 ± 0.04	1.78 ± 0.03	109.08 ± 2.40
E/P-30	25.59 ± 3.39	1.69 ± 0.02	2.07 ± 0.02	94.34 ± 1.84
E/P/F-1	31.40 ± 2.26	1.80 ± 0.04	2.56 ± 0.04	127.01 ± 1.96
E/P/F-3	37.74 ± 0.44	2.07 ± 0.02	2.29 ± 0.02	128.48 ± 1.84
E/P/F-6	31.77 ± 0.86	2.09 ± 0.05	1.90 ± 0.05	122.49 ± 2.88
E/P/F-10	32.00 ± 1.45	2.10 ± 0.01	1.87 ± 0.01	112.37 ± 1.96

The final tensile and flexural data were calculated as the average of five effective measurements.

S6. Morphology and the Toughening Mechanism of Epoxy Resins

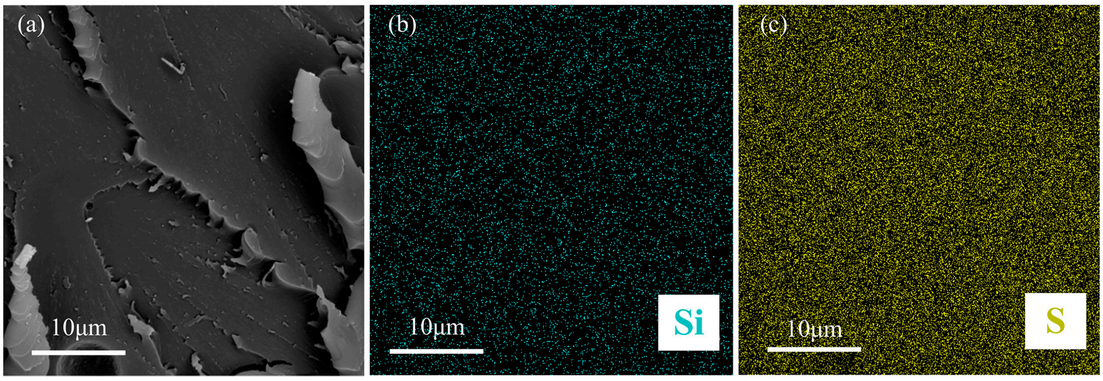

Figure S10. EDS mapping (a-c) of the E/P/F-3 surface.

S7. Thermal Properties of Epoxy Resins

Table S8. Thermal properties of E/P-x and E/P/F-x.

Samples	T_g (℃)	(a)$T_{d5\%}$ (℃)	(a)T_{dmax} (℃)	(a)$Wt_{600℃}$ (%)	(b)$T_{d5\%}$ (℃)	(b)T_{p1} (℃)	(b)T_{p2} (℃)	(b)$Wt_{600℃}$ (%)
EP	137.43	303.99	363.60	18.56	269.36	360.28	485.26	0.93
E/P	103.38	266.87	352.21	18.65	236.00	348.09	491.36	2.52
E/F	141.62	302.60	363.92	19.93	268.72	360.38	485.35	1.63
E/P/F-1	113.31	281.94	349.92	18.97	226.19	364.88	502.52	3.53
E/P/F-3	120.20	276.34	350.91	19.44	240.65	352.03	534.95	3.70
E/P/F-6	114.95	263.98	353.48	20.98	236.05	357.81	532.93	3.94
E/P/F-10	113.01	249.31	353.95	21.34	226.04	351.32	529.82	4.10

(a) and (b) are the results of N_2 and air atmosphere, respectively.

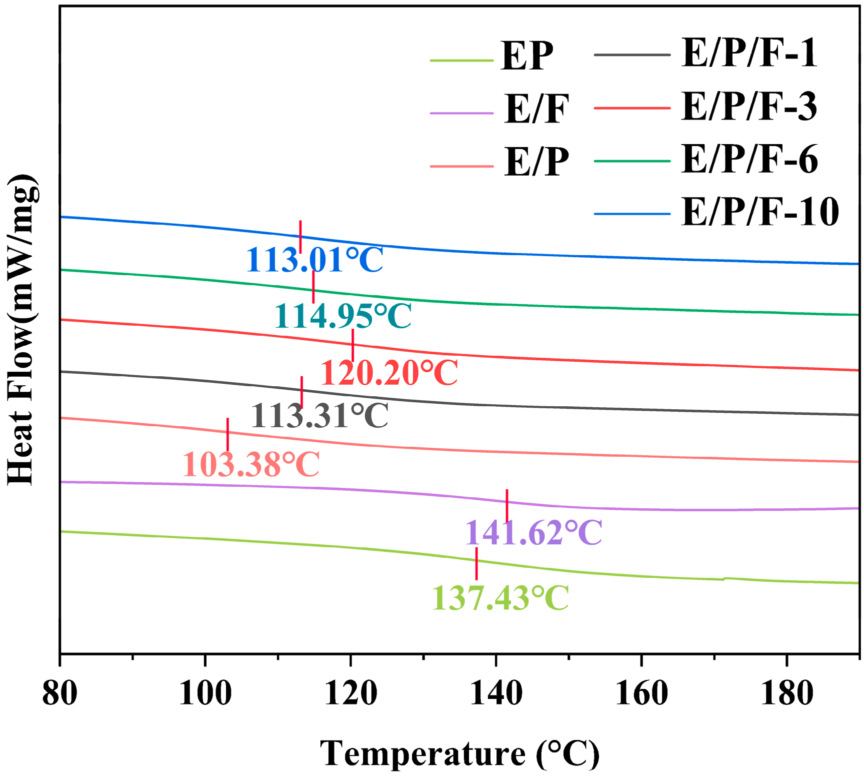

Figure S11. DSC thermograms of EP, E/P, E/F and E/P/F-x hybrids.

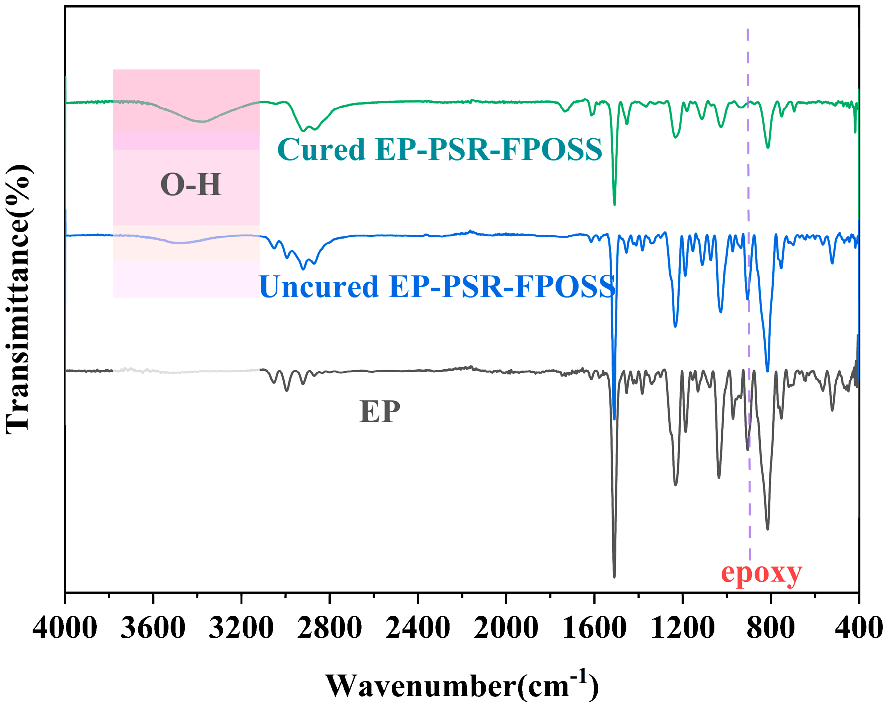

Figure S12. FT-IR spectrum of EP, the uncured and cured hybrids (E/P/F-10)

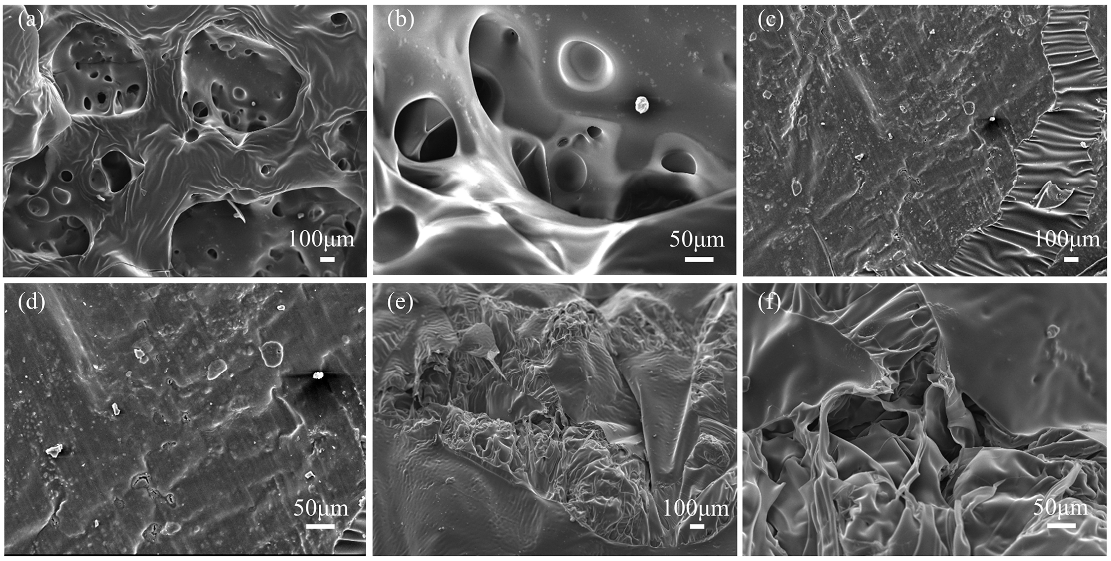

Figure S13. SEM images of the residues: (a) EP at 50×, (b) EP at 200×, (c) the exterior of E/P/F-6 at 50×, (d) the exterior of E/P/F-6 at 200×, (e) the interior of E/P/F-6 at 50×, and (f) the interior of E/P/F-6 at 200×.
